# Supplementary material for: Cell-Free Protein Synthesis by Diversifying Bacterial Transcription Machinery
Source: BioTech (Basel). 2021 Oct 14;10(4):24. doi: 10.3390/biotech10040024 (PMC9245472; doi:10.3390/biotech10040024)
Supplement: Supplementary file 1 [file biotech-10-00024-s001.zip › biotech-1286330-supplementary.pdf]

Supplementary Material

Cell-Free Protein Synthesis by Diversifying Bacterial Transcription Machinery

Marina Snapyan [1*], Sylvain Robin [2], Garabet Yeretssian [1], Michèle Lecocq [1,3], Frédéric Marc [2], Vehary Sakanyan [1,2,3 **]

Table S1. Oligonucleotide primers used for PCR amplification of *T. maritima* genes

Oligonucleotide primer	Sequence starting from 5′ to 3′
Tm Xyl 0808-His	ATGCATCATCATCATCATCATGGAGGGACCCTTTTGAACAT
Tm Xyl 0808-down	CTCGTGATCGGAACTGATGAG
Tm LacI 1856-His	ATGCATCATCATCATCATCATCCAACAATAGAAGATGTCG
Tm LacI 1856-down	GACCACTCGATCTGAACATCC
Tm GntR 0439-His	ATGCATCATCATCATCATCATAAAAAAATCGAAGTGGACCTC
Tm0439-GntR-down	GAACGAAACACCCTCCGCC

Table S2. Oligonucleotide primers used for PCR amplification of promoter-operator regions

Oligonucleotide	Operator DNA	DNA size	Sequence from 5′-extremity
ArgRTn-Up<br>ArgRTn-down	*T. neapolitana argR*	148 bp	IRD700-TGTTACTCTTGAGTTACCAAAAC<br>TTATGAGTTCCTGTCTTC
XylRo-104UP<br>XylRo-down	*E. coli xylFo*	104 bp	IRD800-GGTCATAAATCAAGAAATAAA<br>CACCGCGATAAACGTAACC
LacIo-68UP<br>LacIo-down	*E. coli lacIo*	68 bp	IRD700-GCTTCCGGCTCGTATGT<br>GGTCATAGCTGTTTCCTGTG
GntRo-68UP<br>GntRo-down	*E. coli gntKo*	68 bp	IRD800-GTCCGGCTGGACAATGTT<br>GTGGTGCCCCCACAATAC
ArgCo-UP<br>ArgCo-down	*B. stearothermophilus argCo*	100 bp	CTTAGGGAGGGGCAAGAA<br>CCCGTATGCCTCATGTAG

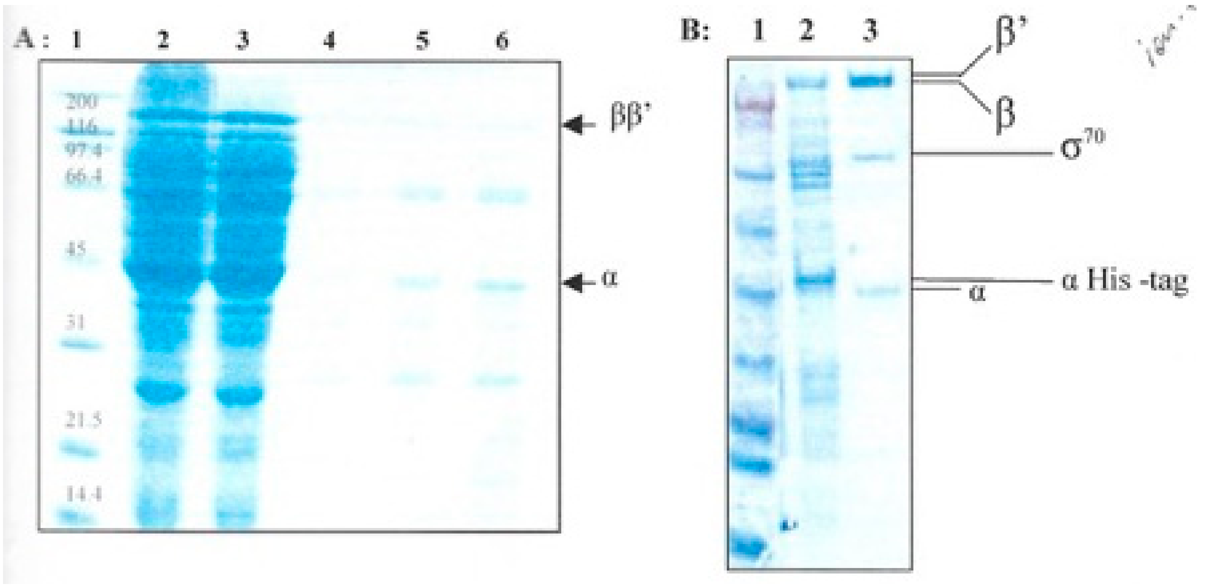

Supplementary Fig. S1. Purification of the core enzyme RNA polymerase from the soluble fraction of *E. coli* extracts. A: 1 - molecular weight marker; 2 and 3 - eluate from a NiNTA column; 4, 5, 6 - various fractions remaining on the column after elution with 40 mM, 65 mM, and 90 mM imidazole. B: 1 - molecular weight marker; 2 - sample containing pooled eluted fractions; 3 - commercial RNA polymerase.

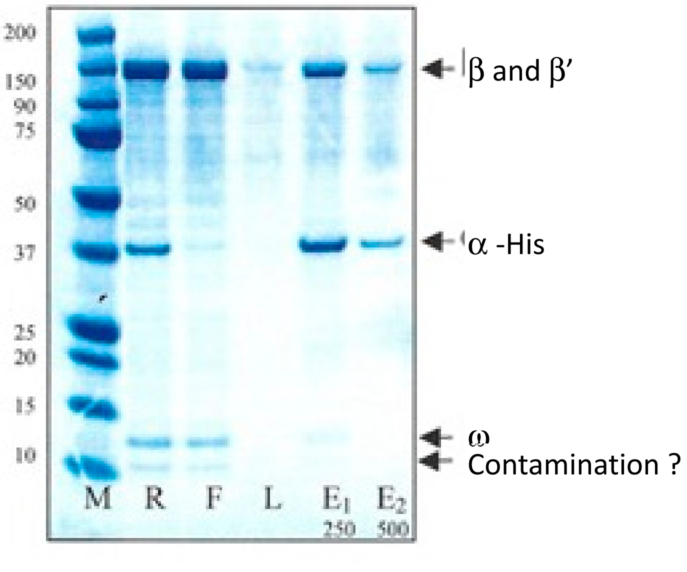

Supplementary Fig. S2. Subunits α, β, β′ and ω of *E. coli* RNA polymerase in inclusion bodies after purification using the His-tagged α subunit. M - molecular mass marker; R - renatured proteins after purification; F - filtrate of proteins not bound on the Ni-NTA column; L - sample washed with 5 mM imidazole; E1 - elution with 250 mM imidazole; E2 - elution with 500 mM imidazole.
